# Efficient non-viral delivery of macromolecules in human primary hematopoietic stem cells and lymphocytes

**DOI:** 10.1093/jmcb/mjad018

**Published:** 2023-03-21

**Authors:** Chuan-Ping Zhang, Hou-Yuan Qiu, Cai-Xiang Zhang, Yu-Ming Zhang, Yi-Zhou Zhang, Hao Yin, Ke-Qin Zhang, Ying Zhang

**Affiliations:** State Key Laboratory for Conservation and Utilization of Bio-Resources in Yunnan, School of Life Sciences, Yunnan University, Kunming 650091, China; Department of Rheumatology and Immunology, Medical Research Institute, Frontier Science Center for Immunology and Metabolism, Zhongnan Hospital of Wuhan University, Wuhan University, Wuhan 430071, China; Department of Rheumatology and Immunology, Medical Research Institute, Frontier Science Center for Immunology and Metabolism, Zhongnan Hospital of Wuhan University, Wuhan University, Wuhan 430071, China; Department of Rheumatology and Immunology, Medical Research Institute, Frontier Science Center for Immunology and Metabolism, Zhongnan Hospital of Wuhan University, Wuhan University, Wuhan 430071, China; Department of Rheumatology and Immunology, Medical Research Institute, Frontier Science Center for Immunology and Metabolism, Zhongnan Hospital of Wuhan University, Wuhan University, Wuhan 430071, China; Department of Rheumatology and Immunology, Medical Research Institute, Frontier Science Center for Immunology and Metabolism, Zhongnan Hospital of Wuhan University, Wuhan University, Wuhan 430071, China; Department of Rheumatology and Immunology, Medical Research Institute, Frontier Science Center for Immunology and Metabolism, Zhongnan Hospital of Wuhan University, Wuhan University, Wuhan 430071, China; State Key Laboratory of Virology, Wuhan University, Wuhan 430071, China; State Key Laboratory for Conservation and Utilization of Bio-Resources in Yunnan, School of Life Sciences, Yunnan University, Kunming 650091, China; Department of Rheumatology and Immunology, Medical Research Institute, Frontier Science Center for Immunology and Metabolism, Zhongnan Hospital of Wuhan University, Wuhan University, Wuhan 430071, China; State Key Laboratory of Virology, Wuhan University, Wuhan 430071, China

**Dear Editor**,

Electroporation (EP) is a non-viral method for introducing macromolecules such as DNA, protein, and messenger RNA (mRNA) into mammalian cells for both basic research and *ex vivo* therapy (Glass et al., 2018). Due to its nature, EP offers great promise as a delivery method for gene therapy without causing risks associated with the viral vector-mediated delivery (Yin et al., 2017; Qiu et al., 2022). EP applies an electrical pulse to permeabilize the cell membrane, so that macromolecules can pass through the pores and diffuse into the cytoplasm (Yarmush et al., 2014). Since membrane proteins vary among cell types, the membrane potential varies; thus, the electric current has to be customized for each cell type. Cell morphology and size can influence the outcome of EP, by affecting cellular conductivity. Cargo properties such as their electric charges also affect EP, requiring different delivery parameters accordingly (Yarmush et al., 2014). Therefore, the optimal EP conditions are highly cell type- and cargo-specific, requiring a careful evaluation.

The CRISPR–Cas9 genome editing system has been widely used due to its simplicity and high efficiency (Hsu et al., 2014). The CRISPR system is composed of the Cas9 protein and a single guide RNA (sgRNA), with the molecular weights of ∼158 kDa and ∼32 kDa, respectively. In cases of gene insertion, a large donor DNA template is also required to be co-delivered into the nucleus of target cells. As of now, efficient gene delivery has been achieved using viral-based vectors (Hsu et al., 2014). Though effective, the risk of insertional mutagenesis, immunogenicity, and limited DNA packaging capacity have hindered broad application of the viral vector-mediated delivery (Taha et al., 2022). Non-viral delivery via EP offers an easy and safe alternative, achieving efficient delivery in mammalian cells. However, in difficult-to-transfect cells such as therapeutically relevant T cells and hematopoietic stem and progenitor cells (HSPCs), relatively low transfection efficiency and high cell toxicity remain an issue to overcome (Yarmush et al., 2014). Lonza 4D-nucleofector™ System is broadly used for EP in mammalian cells. The pre-loaded EP programs paired with the proprietary buffer have been optimized to simplify the experimental setup. However, the unknown formulation of the proprietary buffer restrains further optimization to enhance the EP-mediated non-viral delivery efficiency in primary cell types, particularly the difficult-to-transfect primary cells.

We performed a high-throughput screening of 10 different EP buffers paired with up to 45 EP programs in four different cell lines (Supplementary Figure S1A). By comparing with the commercial standard method (Lonza), we identified several buffer/program combinations showing the improved DNA delivery with >80% efficiency and up to 16-fold increase in median fluorescence intensity (MFI) across four different cell types (Figure 1A). When increasing the electric pulse strength or increasing the electrolyte concentration in the buffer, a higher transfection efficiency can be achieved but comes at the expense of cell viability (Sherba et al., 2020). Under the same electric pulse strength, Jurkat cells in B1mix buffer showed the enhanced viability and improved delivery compared with the cells in Lonza SE buffer (Supplementary Figure S1B). The cells electroporated in B1mix buffer also showed a higher membrane resealing efficiency as revealed by trypan blue staining (Supplementary Figure S1C). Together, these data suggest that the composition of the new buffer is protective against cell toxicity.

**Figure 1:**
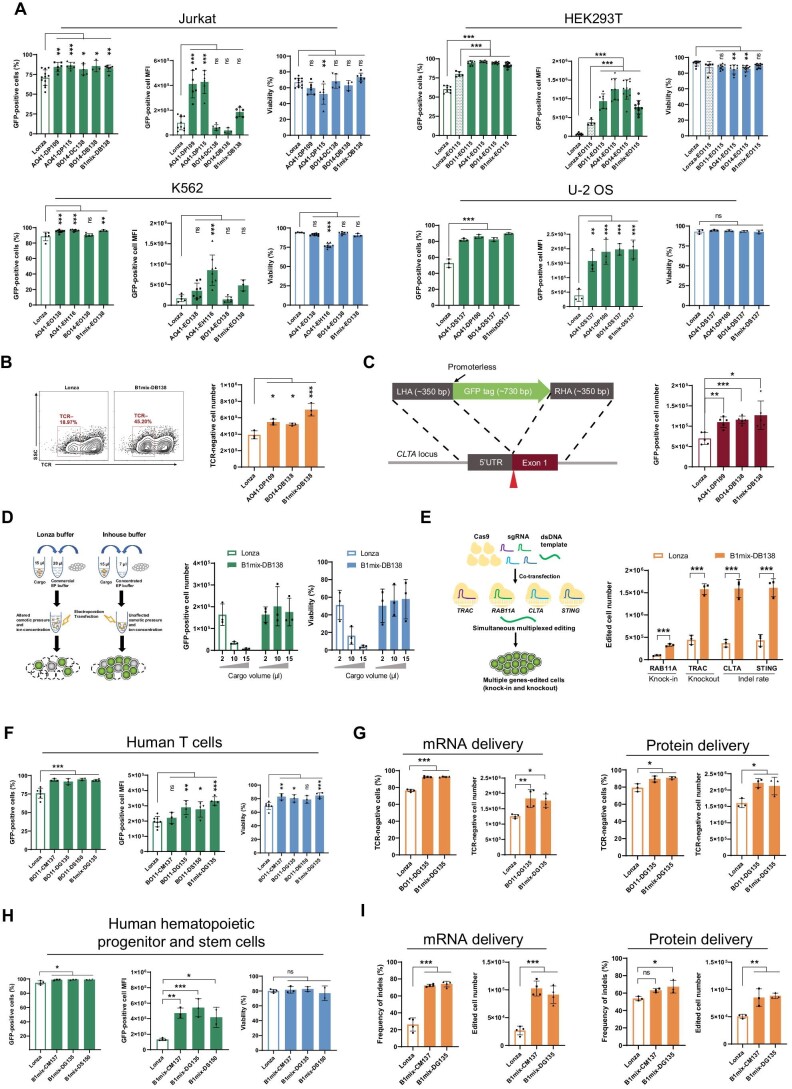
Optimized EP facilitates the delivery of genome editing tools. (**A**) Validation of optimized EP conditions for plasmid delivery in the indicated cell types. Transfection efficiency, MFI in GFP-positive cells, and cell viability were evaluated by FACS. (**B**) FACS plot of TCR knockout using Lonza or optimized EP conditions (left). Optimized EP conditions show increased numbers of TCR knockout cells by plasmid delivery in Jurkat cells (right). (**C**) Illustration of targeted insertion strategy (left) and FACS analysis of absolute GFP knock-in Jurkat cells (right). (**D**) Experimental design testing the effect of cargo volume during EP (left). Volume-dependent decreases in cell viability and the GFP-positive cell number were observed under the Lonza condition, whereas little effect was exhibited under B1mix-DB138 condition (right). (**E**) Multiplex editing strategies for knocking out three genes and knocking in one cassette for GFP expression (left). The edited cell number was calculated by multiplying editing efficiency with the viable cell number (right). (**F** and **H**) Validation of optimized EP conditions for GFP mRNA delivery in primary human T cells (**F**) and HSPCs (**H**). (**G**) Optimized EP conditions improved TCR knockout efficiency using Cas9 mRNA/sgRNA or Cas9/gRNA RNP in primary human T cells. (**I**) Optimized EP conditions increased the frequency of indels and edited cell number using Cas9 mRNA/sgRNA or Cas9/gRNA RNP targeting the *BCL11A* locus in HSPCs. Statistical data were analyzed by one-way analysis of variance. *P < 0.05, **P < 0.01, ***P < 0.001. *n* ≥ 3, mean ± standard deviation.

Cell density can greatly affect EP outcomes by modulating the electric current (Yarmush et al., 2014). The Lonza protocol recommends a tight window of 0.1 million–0.5 million cells in a 20-µl volume. To evaluate the impact of cell density, 0.1 million, 0.5 million, 1 million, 3 million, and 5 million cells were resuspended in 20 µl EP buffer and electroporated. Across five different cell densities, our optimized AO41-DP109 condition exhibited high transfection efficiencies of ∼80% and above, as well as a dose-dependent increase of cell viability (Supplementary Figure S1D), showing the tolerance of a wide cell density range. Only when the cell number increased to 3 million and above, the MFI of GFP-positive cells decreased, indicating that the plasmid concentration may become a rate-limiting factor as the cell number increases. Bovine serum albumin (BSA) and dimethyl sulfoxide (DMSO) have been indicated to improve transfection efficiency or cell viability (Yao et al., 2009; de Menorval et al., 2012).

To test whether it is compatible in our EP system, BSA or DMSO at the indicated dose was added to the EP buffer. Compared with the control, adding BSA or DMSO did not further improve the transfection (Supplementary Figure S1E).

We next determined whether the new protocol can facilitate genome editing. We used CRISPR–Cas9 to disrupt *TRAC* gene encoding T cell receptor (TCR). Since TCR is expressed on Jurkat cells, TCR expression detected by fluorescence-activated cell sorting (FACS) was used to assay the editing efficiency (Figure 1B). The plasmid carrying Cas9 and sgRNA targeting the *TRAC* locus was electroporated under the indicated conditions. Our EP conditions significantly enhanced TCR knockout efficiency, evidenced by both sequencing and FACS analyses (Figure 1B; Supplementary Figure S1F). We further validated the EP conditions in K562 or HEK293T cell line targeting the *TRAC* or *EGFP* locus, respectively. As revealed in Supplementary Figure S1G, new EP conditions generated significantly more edited cells than the Lonza condition. To determine whether the new protocol can mediate targeted gene insertion, we co-transfected the Cas9/gRNA ribonucleoprotein (RNP) complex with a dsDNA template to introduce a GFP fusion protein expression sequence in the housekeeping gene *CLTA* (Figure 1C). Our EP conditions resulted in 1.3- to 3.6-fold increases in GFP-positive cell number compared to the Lonza condition, achieving 16%–43% knock-in efficiencies in Jurkat, K562, U-2 OS, and HEK293T cells (Figure 1C; Supplementary Figure S2A).

The concentration of electrolytes in the EP buffer is critical for transducing correct electric current and allowing efficient cargo delivery (Sherba et al., 2020). The biggest bottleneck of using commercial EP kits is that the cargo volume has to be <10% of the buffer volume, not altering the electric or osmotic property of the buffer. The preparation of concentrated cargo is a rate-limiting step, as some cargos may aggregate at the high concentration. Since our customized buffers have the defined compositions, we attempted to prepare a concentrated buffer allowing larger cargo volumes. We mixed 1 µg plasmid in variable volumes (2, 10, and 15 µl) to 1× Lonza buffer or our B1mix buffer prepared at 3× concentration (Figure 1D). Under the Lonza condition, increasing the cargo volume led to volume-dependent decreases in cell viability and the GFP-positive cell number (Figure 1D). In contrast, the concentrated B1mix buffer allowed the plasmid delivery to be constantly efficient as the cargo volume increased. Similar results were achieved in HEK293T cells (Supplementary Figure S2B), showing little restriction on the cargo volume when using the new buffer. One advantage of the flexible cargo volume is to facilitate multiplex genome editing, which is particularly important in the generation of universal donor cells for cell therapy (Liu et al., 2017; Ren et al., 2017; Ju et al., 2019). As a proof of concept, we attempted to simultaneously knock out three genes (*TRAC, CLTA*, and *STING*) and knock in a GFP expression cassette into the *RAB11A* locus in Jurkat cells (Figure 1E). A large volume of the cargo (15 µl) containing RNP and donor DNA template was added to 20 µl Lonza buffer or 5 µl 3× concentrated B1mix buffer. The concentrated B1mix buffer resulted in an average of 89% knockout efficiency and generated 3-fold more gene knockout cells than the Lonza buffer (Figure 1E). More importantly, B1mix buffer improved cell viability, resulting in 3.6-fold more total viable cells than the Lonza buffer (Supplementary Figure S2C). Together, we demonstrated the superior property of the newly optimized buffer in mediating large-volume (up to 75% of total volume) cargo delivery with high efficiencies and little effect on cell viability.

To determine the optimal EP conditions in therapeutically relevant cell types, such as primary human T cells and HSPCs, which do not tolerant a plasmid transfection well, GFP mRNA was used to screen the buffer/program combinations (Supplementary Figure S2D). The conditions showing superior transfection to the Lonza condition were further validated in human T cells or HSPCs (Figure 1F and H). In primary human T cells, B1mix-DG135 condition exhibited 93.5% transfection efficiency, while the Lonza condition only showed 76.0%. The MFI of GFP-positive cells and cell viability were higher under B1mix-DG135 condition than that under the Lonza condition (Figure 1F). In HSPCs, B1mix buffer resulted in the enhanced transfection efficiency, increased MFI, and comparable cell viability to the Lonza buffer (Figure 1H). Then, the new conditions were used to delivery CRISPR tools targeting therapeutically relevant *TRAC* or *BCL11A* locus in human T cells or HSPCs, respectively. Cas9 mRNA or protein was co-delivered with chemically modified gRNAs. In both human T cells and HSPCs, our optimized EP conditions significantly outperformed the Lonza condition in either Cas9 mRNA or protein delivery (Figure 1G and I), demonstrating that the new B1mix buffer could robustly facilitate the delivery of macromolecules in therapeutically relevant cell types (Supplementary Table S1).

Here, we identified an optimized EP protocol for efficient delivery of DNA, RNA, and protein in multiple cell lines and therapeutically relevant human primary cells. In a side-by-side comparison with the Lonza method, our optimized EP conditions significantly increased not only transfection efficiency but also the MFI of transfected cells. Furthermore, the optimized EP conditions generated significantly more gene knockout or target gene-inserted cells and could deliver protein and mRNA with an equal or higher delivery efficiency, compared with the Lonza condition. Importantly, the new EP conditions also outperformed the Lonza condition in delivering macromolecules in human T cells or HSPCs. It was noted that significantly lower doses of RNP were used under our EP conditions, up to 5-fold less for the *BCL11A* locus in HSPCs (Wu et al., 2019; Demirci et al., 2020; Weber et al., 2020) and ∼10-fold less for the *TRAC* locus in human T cells (Osborn et al., 2016), while maintaining comparable editing efficiencies.

DMSO has been known to enhance cell membrane permeability, thereby facilitating the delivery of small molecules (de Menorval et al., 2012). EP functions to create membrane pores, so that macromolecules can be delivered into the cells. Since our EP conditions were optimized to maximize the generation of resealable membrane pores allowing efficient DNA delivery, DMSO-induced cell permeabilization is likely negligible given the overlapping function. Previous study has suggested that BSA functions to promote cell health in certain EP conditions. When adding BSA or fetal bovine serum into basal RPMI medium, cell viability increased from 20% to 50% (Yao et al., 2009). In our protocol, the basal viability level was 69%, and there were several components to promote membrane resealing and cell health. Due to a high basal level of cell viability and the redundant function, the effect of BSA is also likely negligible.

Multiplex genome editing is particularly important in the generation of universal donor cells for cell therapy. Our newly developed B1mix buffer with a defined composition can be prepared at a higher concentration to allow a larger cargo volume without altering the physical–chemical property of the transfection system. Using the concentrated B1mix buffer, we were able to deliver the cargo in up to 75% of total volume with high cell viability and transfection efficiency, simultaneously achieving ∼89% gene knockout and ∼18% knock-in cells. In sum, our new EP protocol showed superior transfection efficiency, enhanced cell viability, and expanded cargo volume range to the Lonza protocol, representing a straightforward and robust approach for transient gene expression and gene modification of cell lines and human primary cells.

*We thank the core facility of Medical Research Institute at Wuhan University for their technical supports. This work is kindly supported by the National Key Research and Development Program of China (2022YFF1002801 to Y.Z., 2019YFA0802801 and 2018YFA0801401 to H.Y.), the National Natural Science Foundation of China (31972936 to Y.Z.), Medical Science Advancement Program (Basic Medical Sciences) of Wuhan University (TFJC2018005), the Fundamental Research Funds for the Central Universities (to Y.Z.), and the startup funding from Wuhan University (to Y.Z.). Y.Z., H.Y., C.-P.Z., and J.A. have filed a patent application on buffer composition through Wuhan University (PCT International Application No. PCT/CN2022/106565). Y.Z. conceived, designed, and managed the project with support from K.-Q.Z.; C.-P.Z. designed, performed experiments, and analyzed data; H.-Y.Q. and Y.-M.Z. helped with experiments; C.-X.Z. and Y.-Z.Z. drew the heatmap; C.-P.Z. and Y.Z. wrote the paper with input from H.Y.]*

## Supplementary Material

mjad018_Supplemental_File
