# Supplementary material for: Efficient non-viral delivery of macromolecules in human primary hematopoietic stem cells and lymphocytes
Source: J Mol Cell Biol. 2023 Mar 21;15(3):mjad018. doi: 10.1093/jmcb/mjad018 (PMC10481097; doi:10.1093/jmcb/mjad018)
Supplement: mjad018_Supplemental_File [file mjad018_supplemental_file.pdf]

Supplementary material

Efficient non-viral delivery of macromolecules in human primary hematopoietic stem cells and lymphocytes

Chuan-Ping Zhang[1,2], Hou-Yuan Qiu[2], Cai-Xiang Zhang[2], Yu-Ming Zhang[2], Yi-Zhou Zhang[2], Hao Yin[2,3], Ke-Qin Zhang[1,*], and Ying Zhang[2,3,*]

[1] State Key Laboratory for Conservation and Utilization of Bio-Resources in Yunnan, School of Life Sciences, Yunnan University, Kunming 650091, China

[2] Department of Rheumatology and Immunology, Medical Research Institute, Frontier Science Center for Immunology and Metabolism, Zhongnan Hospital of Wuhan University, Wuhan University, Wuhan 430071, China

[3] State Key Laboratory of Virology, Wuhan University, Wuhan 430071, China

* Correspondence to: Ying Zhang, E-mail: ying.zhang84@whu.edu.cn; Ke-Qin Zhang, E-mail: kqzhang1@ynu.edu.cn

Supplementary Material and methods

Plasmids and cloning

The pMAX GFP plasmid (3.5 kb) was purchased from the Lonza 4D-Nucleofector X Kit (Lonza). 508 GFP plasmid (8.5 kb) was a common EGFP expression plasmid constructed by inserting CMV and EGFP sequences into the plasmid skeleton. CRISPR plasmid pX330 (#42230) encoding *S. pyogenes* Cas9 and the U6 promoter was purchased from Addgene. gRNAs targeting T-cell receptor α constant (*TRAC*) or enhanced green fluorescent protein (*EGFP*) was cloned in BbsI-digested pX330 backbone. gRNA sequences used in this study are listed in Supplementary Table S2.

gRNA and RNP complex preparation

gRNA was generated via *in vitro* transcription: DNA template for *in vitro* transcription was generated by PCR amplification with primers containing T7 promotor and gRNA sequence (Supplementary Table S2). Approximately 0.5–1 µg DNA was used for *in vitro* transcription per 50 µl reaction and the reaction was carried out by adding 2 µl T7 RNA polymerase (15 U/µl), 4 µl rNTP mix (25 mM), 5 µl DTT (50 mM), 4 µl PPase (25 U/ml), and 1.25 µl RNase Inhibitor (40 U/µl) at 37°C for 1.5 h. DNase I was used to digest DNA template

and the products were purified using the NEB Monarch® RNA Cleanup Kit (NEB, T2040) and quantified by Nanodrop (Thermo Fisher). The purity of gRNA was measured in 10% urea-PAGE. *In vitro* transcribed gRNA was treated with alkaline phosphatase calf Intestinal (CIP) to remove the 5'-triphosphate, avoiding immunogenic reaction in primary cells (Kim et al., 2018). After adding 0.5 U CIP (NEB, M0290L) and 5 U RNase inhibitor (Promega, N2615) per 1 μg sgRNA according to the manufactory's instructions, the reaction was carried out in 10× reaction buffer at 37°C for 3 h. After CIP treatment, sgRNA was purified with RNA Cleanup Kit again. Chemically modified sgRNAs, with 2'-O-methyl and phosphorothioate modifications at the first three 5'- and 3'-terminal RNA residues (Hendel et al., 2015), were purchased from Genscript. Cas9 protein was purchased from Aldevron. RNPs were prepared by mixing Cas9 protein with sgRNA at a 1:5 molar ratio and incubated at room temperature for at least 10 min.

Cell lines and culture conditions

Jurkat and K562 cells were purchased from ATCC and cultured in RPMI1640 medium (Gibco) supplemented with 10% FBS (AusGeneX) and 1% Penicillin/Streptomycin (Gibco). HEK293T and U-2 OS cells were obtained from CCTCC and cultured in DMEM (Gibco) supplemented with 10% FBS and 1% Penicillin/Streptomycin. HEK293T-EGFP cell line was generated by infecting HEK293T17 with lentivirus carrying a EGFP construct. The cells were cultured at 37°C, 5% CO_2, and were subcultured every 2 to 3 days.

Primary cells and culture conditions

Primary human T cells

Human primary T cells were isolated from whole blood (SAILY BIO) of healthy donors. Peripheral blood mononuclear cells (PBMCs) were isolated by Ficoll Paque Plus (GE, 17144003) density centrifugation in SepMate tube (STEMCELL). $CD3^+$ T cells were isolated from PBMCs using EasySep Human T Cell Isolation Kit (STEMCELL) following the manufactory's instructions. Isolated T cells were adjusted to 0.5–1×10^6/ml in the medium, and CD3/CD28 dynabeads (Thermo Fisher) were added to activate T cells for 3 days. T cells were cultured in X-Vivo15 medium (Lonza) supplemented with 5% FBS (Gibco), 10 ng/ml IL-7, 50 ng/ml human IL-2 (Peprotech), and 1% Penicillin/Streptomycin. After 3 days of activation, human T cells were cultured in medium with IL-2 at 100 ng/ml.

$CD34^+$ hematopoietic stem and progenitor cells (HSPCs)

Mobilized peripheral blood-derived $CD34^+$ HSPCs (SAILY BIO) were thawed according

to manufacturer's instructions. The HSPCs were cultured in StemSpan SFEM (STEMCELL) supplemented with 100 ng/ml recombinant human Flt3 ligand, 100 ng/ml human thrombopoietin, 100 ng/ml human stem cell factor (Peprotech), and 1% Penicillin/Streptomycin (Gibco). HSPCs were cultured at 37°C, 5% CO_2, and the medium was changed every 2 days.

HDR template production

Reported HDR template sequences were used in the study (Supplementary Table S2). The insert fragment and tow homologous arms and insert fragments of the HDR template were amplified by PCR, respectively. All fragments of HDR template were constructed into PUC57 cloning vector ligated by Gibson assembly (TransGen Biotech) into a cloning vector for sequence validation and future propagation. Approximately 1 ng HDR donor plasmid was used as PCR template per 50 μl reaction and PCR products were column-purified by PCR purification Kit (ZYMO, D4034) to prepare dsDNA HDR template. The purity and size of the amplified HDR template were confirmed by agarose gel (1.0%) electrophoresis. DNA concentration was measured using nanodrop (Thermo Fisher).

Electroporation buffer

Basic buffer B (15 mM $MgCl_2$, 5 mM KCl, 25 mM sodium succinate, 25 mM Manitol, 120 mM Na_2HPO_4/NaH_2PO_4, pH 7.2) and Basic buffer A (15 mM $MgCl_2$, 5 mM KCl, 90 mM NaCl, 0.4 mM $Ca(NO_3)_2$, 10 mM glucose, 40 mM Na_2HPO_4/NaH_2PO_4, pH 7.2) were prepared according to the reported study (Chicaybam et al., 2016). Basic buffer O is Opti-MEM medium (Thermo Fisher) with pH 7.2. Basic buffer B1 is buffer B with reduced sodium succinate (13.5 mM). The prepared basic buffer was filtered with 0.2-μm filter membrane and buffer A or buffer B was mixed with buffer O at different ratios to obtain buffer BO14, BO11, and AO41. For example, buffer BO14 was prepared by mixing basic buffer B and buffer Opti-MEM in a 1:4 volume ratio. Buffer B1mix was prepared by mixing 72.3% basic buffer B1 with five important components (0.5 mM sodium pyruvate, 0.8 mM $Ca(NO_3)_2$, 0.26 mM i-inositol, 2% GlutaMAX, and 20 mM D-glucose) from Opti-MEM. The prepared buffer can be stored at 4°C for >6 months.

Electroporation

For cell lines, cells in the logarithmic growth phase were collected and electroporated using 4D-nucleofector (Lonza). Prior to electroporation (EP), cells were centrifuged at 90× *g* for 10 min, resuspended in 20 μl of the indicated EP buffer, and mixed with DNA, RNA, or

RNP. Cell mixture was transferred to a 20-μl EP cuvette and electroporated under the indicated EP program. For human primary T cells, 0.6 million cells were electroporated under indicated conditions. For human CD34$^+$ HSPCs, cryopreserved cells were resuscitated and then recovered in the culture medium for 48 h and 0.1 million cells were used for EP. After EP, 85 μl of pre-warmed medium was transferred to each cuvette and cells were placed at 37°C in the incubator for 10–15 min before transferring to culture plate. In HDR experiment, 0.5–1 μg template DNA was added to the RNP complex, and cells were added to the mixture later on. The recommended Lonza EP protocol for each cell type is listed in Supplementary Table S1.

Flow cytometry

Agilent NovoCyte Flow Cytometer was used to perform GFP expression analysis, evaluation of viability, and absolute cell count. Cells were harvested after 1 day for delivery of GFP-plasmid, after 3 days for HDR analysis, and after 7 days for EGFP knockout efficiency analysis. To determine surface protein expression, 1×10^5 cells were stained with APC-TCRα/β antibodies (Biolegend) for 20 min on ice. The stained cells were measured on a NovoCyte flow cytometer and analyzed with Novo Express Software. Indel rate of edited gene loci was evaluated by TIDE analysis using the TIDE web tool (Brinkman et al., 2014).

Statistical analyses

GraphPad Prism 8 was used for statistical analysis. Student's *t*-test and one-way ANOVA analysis were used to determine statistical significance.

Supplementary References

Brinkman, E.K., Chen, T., Amendola, M., et al. (2014). Easy quantitative assessment of genome editing by sequence trace decomposition. Nucleic Acids Res. *42*, e168.

Chicaybam, L., Barcelos, C., Peixoto, B., et al. (2016). An efficient electroporation protocol for the genetic modification of mammalian cells. Front. Bioeng. Biotechnol. *4*, 99.

Hendel, A., Bak, R.O., Clark, J.T., et al. (2015). Chemically modified guide RNAs enhance CRISPR–Cas genome editing in human primary cells. Nat. Biotechnol. *33*, 985–989.

Kim, S., Koo, T., Jee, H.G., et al. (2018). CRISPR RNAs trigger innate immune responses in human cells. Genome Res. *28*, 367–373.

Supplementary Figures

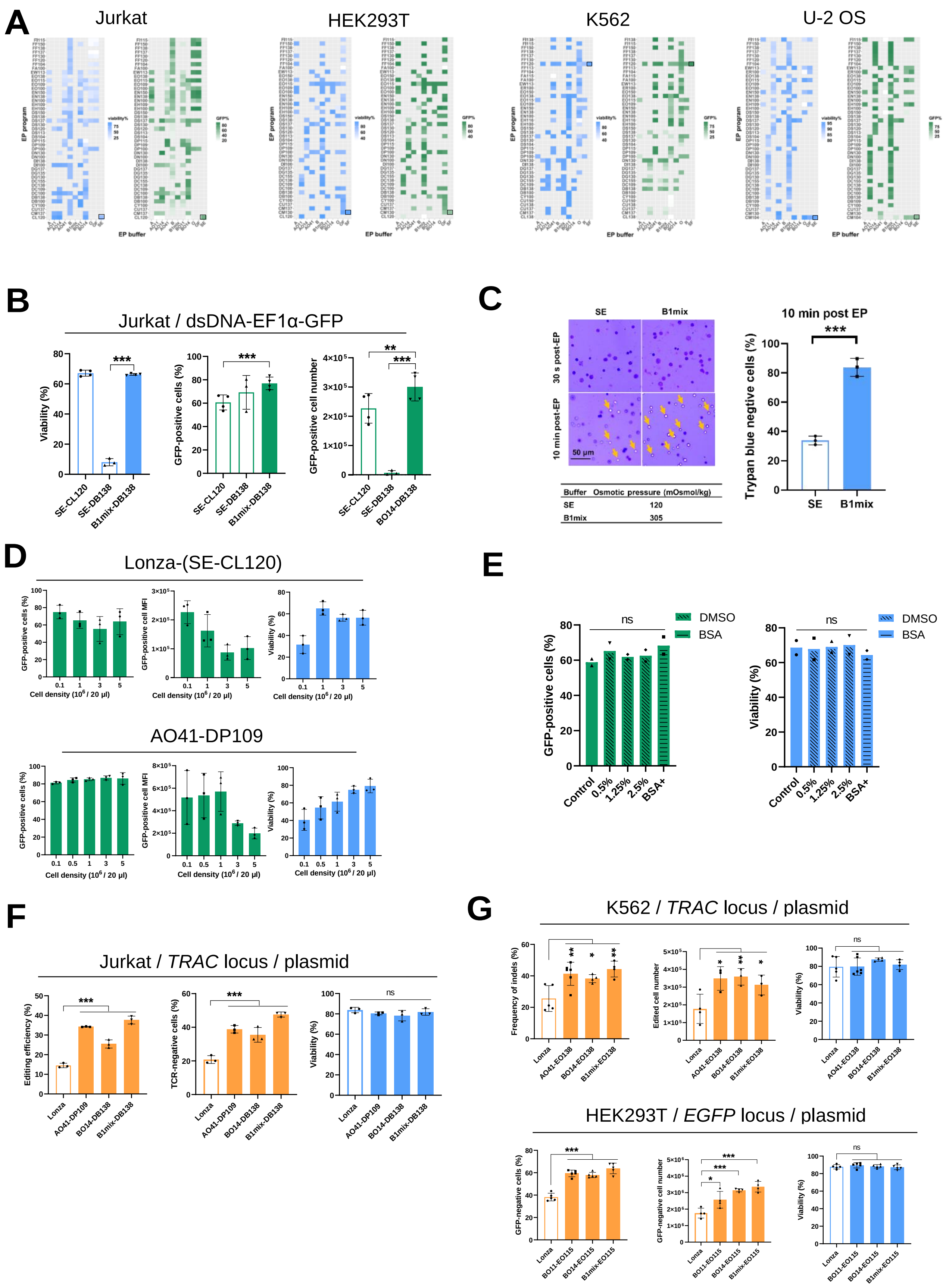

Supplementary Figure S1 High-throughput screening of EP conditions identifies new buffers with enhanced delivery of CRISPR tools in multiple cell types. (**A**) Heatmap of transfection efficiency and viability. Approximately 0.5 μg pMAX GFP plasmid was electroporated in cell lines. Lonza recommended condition was included and boxed black. Data were from one experiment. (**B**) Comparison of dsDNA delivery efficiencies and cell viabilities in Jurkat cells based on buffer B1mix and commercial buffer SE. (**C**) Comparison of membrane resealing efficiencies post-EP. Trypan blue staining was used to detect membrane resealing at 30 sec or 10 min post-EP in Jurkat cells. (**D**) Variable cell densities were tested in Lonza commercial EP condition or in newly developed EP condition. Approximately 0.5 μg pMAX plasmid was used. (**E**) FACS analysis of transfection efficiency (**left**) and viability (**right**) using EP buffers supplemented with DMSO or BSA. (**F** and **G**) Optimized EP conditions show increased CRISPR-mediated gene knockout using plasmid in Jurkat (**F**) and K562 or HEK293T cells (**G**). Approximately 1 μg pX330 plasmid was used. *P*-value was calculated by one-way ANOVA for the entire figure. *$P < 0.05$, **$P < 0.01$, ***$P < 0.001$, $n \geq 3$ biologically independent samples. Error bars represent mean ± SD.

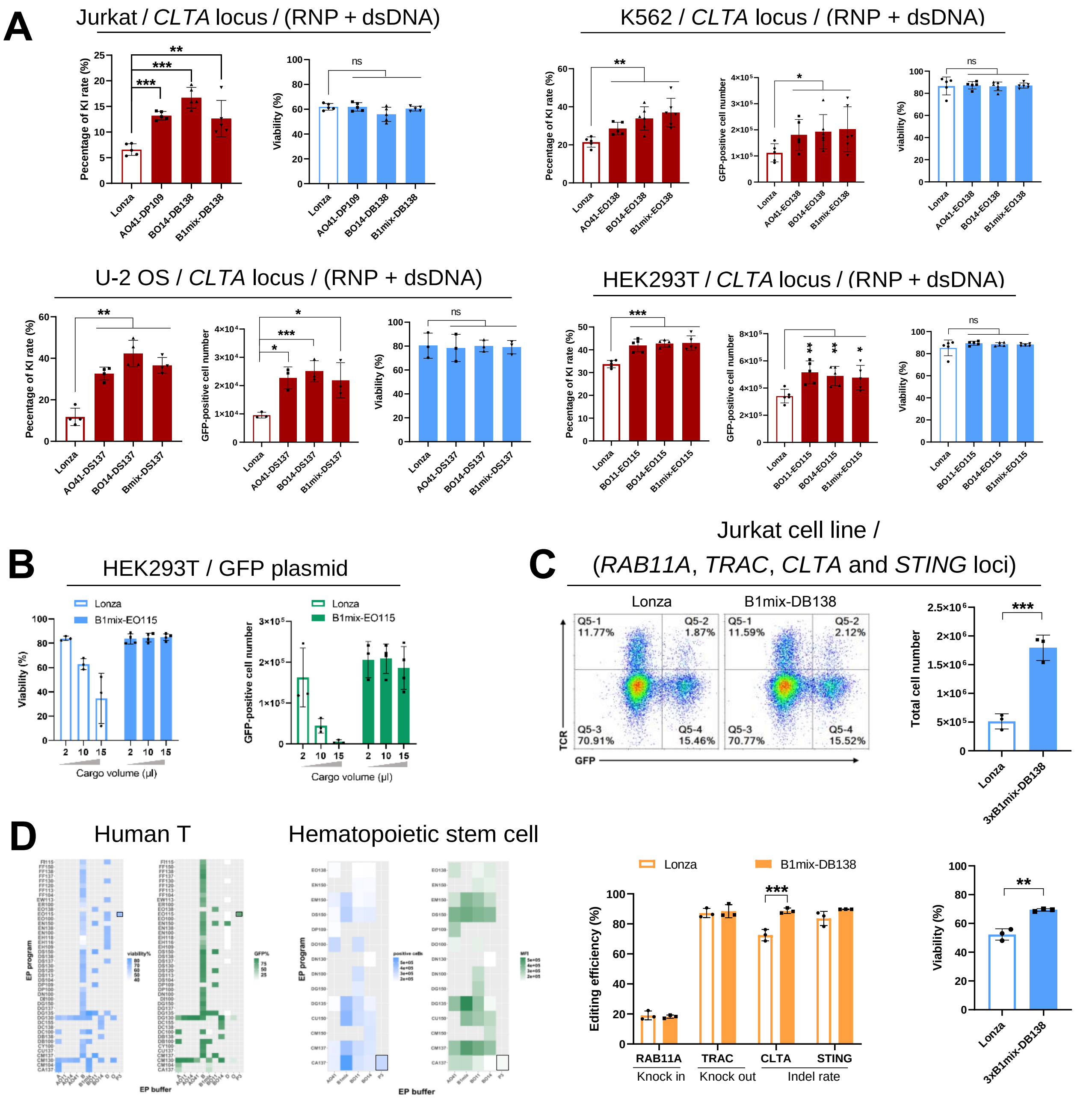

Supplementary Figure S2 Optimized EP achieves higher target insertion efficiency and allows larger cargo volumes during EP. (**A**) FACS analysis of GFP knock-in efficiency and viability in Jurkat, K562, U-2 OS, and HEK293T cell lines by co-EP of RNP and dsDNA template under indicated conditions. (**B**) Cell viability (**left**) and GFP-positive cell number (**right**) exhibited volume-dependent decrease when using Lonza condition whereas little effect was observed using customized buffers. Approximately 1 μg GFP-plasmid was used and data were collected at 24 h post-EP. (**C**) FACS and indel analysis of multiplex editing outcome using Lonza or customized concentrated buffer condition. (**D**) Heatmap of transfection efficiency and viability. Approximately 0.5 μg GFP mRNA was electroporated in primary cells. Lonza recommended condition was included and boxed black. Data were from one experiment. *P*-value was calculated by one-way ANOVA. *$P < 0.05$, **$P < 0.01$, ***$P < 0.001$, n ≥ 3 biologically independent samples. Error bars represent mean ± SD.

Supplementary Table S1 Optimized EP conditions for each cell type.

Cell	Cell type	EP buffer	Recommended program	GFP (%) (new condition)	GFP (%) (Lonza)	GFP-positive cell MFI (new condition)	GFP-positive cell MFI (Lonza)	Viability (%) (new condition)	Viability (%) (Lonza)
**Primary cells**									
Human T	Human primary T cell	B1mix	DG135	93.49±1.28	76.04±6.72	3.31±0.28 ($\times10^5$)	1.96±0.34 ($\times10^5$)	84.54±4.29	69.38±5.26
HSPC	hematopoietic stem and progenitor cell	B1mix	DG135	99.38±0.92	94.72±2.58	5.41±1.17 ($\times10^5$)	1.32±0.14 ($\times10^5$)	82.69±3.22	80.01±2.26
**Cell lines**									
Jurkat	Acute T cell leukemia, T lymphocyte	B1mix/AO41/BO14	DB138	82.82±4.17	70.77±9.91	1.85±0.39 ($\times10^5$)	0.97±0.55 ($\times10^5$)	73.41±5.02	66.76±5.34
K562	Human chronic myelogenous leukemia	AO41/B1mix	EO138	95.16±1.76	88.54±5.55	3.48±1.92 ($\times10^5$)	1.70±0.83 ($\times10^5$)	91.42±1.23	94.01±0.45
HEK293T	Human embryonal kidney	AO41/BO14/B1mix	EO115	80.09±3.24	60.39±4.22	9.35±1.95 ($\times10^5$)	0.64±0.29 ($\times10^5$)	88.93±3.28	92.49±3.26
U-2 OS	Human bone osteosarcoma epithelial cell	AO41	DS137	81.93±1.75	52.58±5.27	1.58±0.37 ($\times10^5$)	0.38±0.21 ($\times10^5$)	94.58±0.83	92.84±2.63

Supplementary Table S2 gRNAs and HDR templates used in this study.

Name	Sequence
**gRNA-EGFP**	GGGCGAGGAGCTGTTCACCG
**gRNA-TRAC**	CAGGGTTCTGGATATCTGT
**gRNA-STING**	GCTGGGACTGCTGTTAAACG
**gRNA-RAB11A**	GGTAGTCGTACTCGTCGTCG
**gRNA-CLTA**	GAACGGATCCAGCTCAGCCA
**CLTA-GFP** (HDR template used in Figure 1C and Supplementary Figure S2A)	CTCCCTATCAGGCAGCACTTCCGCCTCCCGGGGCCCGCGCAGCTCACCTCCCTCACCTCCCGCCCTACCCCAGTCACGAGTTGTTTTAGGGGGACCGCCCCTCCACTTGCTGATTGGGTAGCTCCTGAACCATTGTTGTCCTCTGATTGGTTGTTCCCTTTTCGGCTCTGCAACACCGCCTAGACCGACCGGATACACGGGTAGGGCTTCCGCTTTACCCGTCTCCCTCCTGGCGCTTGTCCTCCTCTCCCAGTCGGCACCACAGCGGTGGCTGCCGGGCGTGGTGTCGGTGGGTCGGTTGGTTTTTGTCTCACCGTTGGTGTCCGTGCCGTTCAGTTGCCCGCCATGGCTGGATCGGGTGGGACTAGTGGCAGCAAGGGCGAGGAGCTGTTCACCGGGGTGGTGCCCATCCTGGTCGAGCTGGACGGCGACGTAAACGGCCACAAGTTCAGCGTGCGCGGCGAGGGCGAGGGCGATGCCACCAACGGCAAGCTGACCCTGAAGTTCATCTGCACCACCGGCAAGCTGCCCGTGCCCTGGCCCACCCTCGTGACCACCCTGACCTACGGCGTGCAGTGCTTCAGCCGCTACCCCGACCACATGAAGCGCCACGACTTCTTCAAGTCCGCCATGCCCGAAGGCTACGTCCAGGAGCGCACCATCAGCTTCAAGGACGACGGCACCTACAAGACCCGCGCCGAGGTGAAGTTCGAGGGCGACACCCTGGTGAACCGCATCGAGCTGAAGGGCATCGACTTCAAGGAGGACGGCAACATCCTGGGGCACAAGCTGGAGTACAACTTCAACAGCCACAACGTCTATATCACCGCCGACAAGCAGAAGAACGGCATCAAGGCCAACTTCAAGATCCGCCACAACGTGGAGGACGGCAGCGTGCAGCTCGCCGACCACTACCAGCAGAACACCCCCATCGGCGACGGCCCCGTGCTGCTGCCCGACAACCACTACCTGAGCACCCAGTCCGTGCTGAGCAAAGACCCCAACGAGAAGCGCGATCACATGGTCCTGCTGGAGTTCGTGACCGCCGCCGGGATCACTGGAACCGGTGCTGGAAGTGGTGAGCTGGATCCGTTCGGCGCCCCTGCCGGCGCCCCTGGCGGTCCCGCGCTGGGGAACGGAGTGGCCGGCGCCGGCGAAGAAGACCCGGCTGCGGCCTTCTTGGCGCAGCAAGAGAGCGAGATTGCGGGCATCGAGAACGACGAGGCCTTCGCCATCCTGGACGGCGGCGCCCCCGGGCCCCAGCCGCACGGCGAGCCGCCGGGGGGTCCGGGTGAGAGTGCGGGCGCGTTTGGGGCGAGAGGACTTGTCTGGAAACTCGGTCCACAGTGGGTCCGAGAGCTTCTGTGTGACTCGTGCTCCTTG
**RAB11A-GFP** (HDR template used in Figure 1E and Supplementary Figure S2C)	GGTAGCTAGGAGTTCCAGGACTCAGTTTCCCCTTTGAGCCTCCTTTAGCGACTAAAGCTTGAAGCCCCACGCATCTCGACTCTCGCGCACACCGCCCTTGTTGGGCTCAGGGGCGGGGCGCCGCCCCCGGAAGTACTTCCCCTTAAAGGCTGGGGCCTGCCGGAAATGGCGCAGCGGCAGGGAGGGGCTCTTCACCCAGTCCGGCAGTTGAAGCTCGGCGCTCGGGTTACCCCTGCAGCGACGCCCCCTGGTCCCACAGATACCACTGCTGCTCCCGCCCTTTCGCTCCTCGGCCGCGCAATGGGCGGATCGGGTGGGACTAGTGGCAGCAAGGGCGAGGAGCTGTTCACCGGGGTGGTGCCCATCCTGGTCGAGCTGGACGGCGACGTAAACGGCCACAAGTTCAGCGTGCGCGGCGAGGGCGAGGGCGATGCCACCAACGGCAAGCTGACCCTGAAGTTCATCTGCACCACCGGCAAGCTGCCCGTGCCCTGGCCCACCCTCGTGACCACCCTGACCTACGGCGTGCAGTGCTTCAGCCGCTACCCCGACCACATGAAGCGCCACGACTTCTTCAAGTCCGCCATGCCCGAAGGCTACGTCCAGGAGCGCACCATCAGCTTCAAGGACGACGGCACCTACAAGACCCGCGCCGAGGTGAAGTTCGAGGGCGACACCCTGGTGAACCGCATCGAGCTGAAGGGCATCGACTTCAAGGAGGACGGCAACATCCTGGGGCACAAGCTGGAGTACAACTTCAACAGCCACAACGTCTATATCACCGCCGACAAGCAGAAGAACGGCATCAAGGCCAACTTCAAGATCCGCCACAACGTGGAGGACGGCAGCGTGCAGCTCGCCGACCACTACCAGCAGAACACCCCCATCGGCGACGGCCCCGTGCTGCTGCCCGACAACCACTACCTGAGCACCCAGTCCGTGCTGAGCAAAGACCCCAACGAGAAGCGCGATCACATGGTCCTGCTGGAGTTCGTGACCGCCGCCGGGATCACTGGAACCGGTGCTGGAAGTGGTACACGCGACGACGAGTACGACTACCTCTTTAAAGGTGAGGCCATGGGCTCTCGCACTCTACACAGTCCTCGTTCGGGGACCCGGGCCACTCCCGGTGGACCCTCGTGCCGGCCACCCCTGCACTGATATAGGCCTCCCTCAGCCCTTCCTTTTTGTGCGGTTCCGTCTCCTACCCAGCTCAGCCTCTTCTCCCCCGCTCAGACAGGGGTCCCCATCACATGCCGCTCTCTGAGCGACCTCTCCATAGGCCTTCGCTGGCCTCAGAGCCCCTCCCTGCGTGTCCTTCCCCTGGCGGACTGCCTTCTCCCACATCGT
